# Rapid Prediction of Nutrient Concentration in Citrus Leaves Using Vis-NIR Spectroscopy

**DOI:** 10.3390/s23146530

**Published:** 2023-07-19

**Authors:** Maylin Acosta, Ana Quiñones, Sandra Munera, José Miguel de Paz, José Blasco

**Affiliations:** 1 Centro para el Desarrollo de la Agricultura Sostenible, Instituto Valenciano de Investigaciones Agrarias (IVIA), CV-315, km 10.7, 46113 Moncada, Valencia, Spain; 2 Departamento de Ingeniería Gráfica, Universitat Politècnica de València, Camino de Vera, s/n, 46022 Valencia, Valencia, Spain; 3 Centro de Agroingeniería, Instituto Valenciano de Investigaciones Agrarias (IVIA), CV-315, km 10.7, 46113 Moncada, Valencia, Spain

**Keywords:** citrus nutrition, agricultural sensors, fertilisation, ionomics, chemometrics

## Abstract

The nutritional diagnosis of crops is carried out through costly foliar ionomic analysis in laboratories. However, spectroscopy is a sensing technique that could replace these destructive analyses for monitoring nutritional status. This work aimed to develop a calibration model to predict the foliar concentrations of macro and micronutrients in citrus plantations based on rapid non-destructive spectral measurements. To this end, 592 ‘Clementina de Nules’ citrus leaves were collected during several months of growth. In these foliar samples, the spectral absorbance (430–1040 nm) was measured using a portable spectrometer, and the foliar ionomics was determined by emission spectrometry (ICP-OES) for macro and micronutrients, and the Kjeldahl method to quantify N. Models based on partial least squares regression (PLS-R) were calibrated to predict the content of macro and micronutrients in the leaves. The determination coefficients obtained in the model test were between 0.31 and 0.69, the highest values being found for P, K, and B (0.60, 0.63, and 0.69, respectively). Furthermore, the important P, K, and B wavelengths were evaluated using the weighted regression coefficients (BW) obtained from the PLS-R model. The results showed that the selected wavelengths were all in the visible region (430–750 nm) related to foliage pigments. The results indicate that this technique is promising for rapid and non-destructive foliar macro and micronutrient prediction.

## 1. Introduction

Citrus is one of the most popular and widespread fruit crops worldwide. According to FAO, the world production of citrus fruits was estimated at 152 million tons in 2020. Oranges are the most widely produced citrus fruit worldwide (50.5% of the total), followed by mandarins (33.7%), lemons (8.4%), and grapefruit (7.4%) [1]. The leading citrus producer is China (32.7 million tons), followed by Brazil (16.6 million tons), India (9.8 million tons), and the United States (7.8 million tons). Spain, with a cultivated surface area of 300,504 ha, reaches a production of 6.8 million tons and is the first producer country in the EU, followed by Italy [2]. Moreover, Spain is the foremost global provider of fresh citrus fruits, commanding approximately 25% of the export market worldwide [3].

This high level of production requires optimal handling of resources. Among the primary agricultural inputs are those related to fertilisation management, such as nutrients. Nutrients are essential elements for the growth and productivity of crops and can be categorised as macronutrients and micronutrients based on the relative amounts required by plants [4]. Reducing unnecessary fertiliser use helps to reduce costs, improve fruit quality and minimise the risk of contamination. Excess or nutritional deficiency can affect vegetative development and crop yield. Sixteen elements are considered essential nutrients for the optimum development of crops. These nutrients include carbon (C), oxygen (O), hydrogen (H), nitrogen (N), phosphorus (P), potassium (K), calcium (Ca), magnesium (Mg), sulphur (S), iron (Fe), manganese (Mn), zinc (Zn), copper (Cu), boron (B), molybdenum (Mo), chlorine (Cl), and nickel (Ni). Plants absorb C and O from the air through the leaves as carbon dioxide (CO_2_). The photosynthesis process transforms CO_2_ and water into H, C, and O. The other nutrients are absorbed through the root system or foliar surface and must be supplied during the growth cycle following fertilisation plans [5].

Macronutrients are essential elements for plants and are required in relatively large amounts. Nitrogen is a major factor in photosynthesis since chlorophyll molecules contain this nutrient [6,7], and it is the nutrient that affects, to a greater extent, the vegetative development of the plant. Phosphorus intervenes in the transport, storage and transfer of energy, stimulating root development and favouring the flowering and fruit set. Potassium is the element that influences production the most since the fruit is the main sink of this nutrient. Calcium plays a crucial role in activating and regulating various cellular processes, including cell division and elongation. It also influences the organisation of cells, particularly concerning the specialisation of cell organelles and the translocation of carbohydrates. Magnesium is part of the chlorophyll molecule (photosynthesis), a constituent of cell walls, and plays a vital role in P translocation and N assimilation.

Regarding micronutrients, S is the key to protein synthesis, a component of sulphur-containing AA (cysteine and methionine) and is part of vitamins and coenzymes. Iron deficiency is common in calcareous soils. It is an essential element for the growth and development of plants since it participates in numerous enzymatic and metabolic processes and in the synthesis of chlorophyll. Zinc is part of the chlorophyll molecule (photosynthesis), and Mn is bound to Fe for chlorophyll formation (photosynthesis). Cooper is involved in photosynthesis and carbohydrate metabolism. Boron is necessary for lignin biosynthesis, involving cell division and root elongation [8]. Molybdenum is a key component in two enzymes that convert nitrate to nitrite and ammonia. Its absence prevents the correct transformation of N into amino acids. Chloride is essential for plant growth but is absorbed by plants in minimal quantities. And finally, Ni is necessary for N metabolism and plant germination.

Therefore, nutritional diagnosis is essential for efficient fertilisation management, especially at the early stages of crop development when it influences the production quality. Traditionally, the nutritional status of plants is determined via a leaf ionomic analysis carried out in a laboratory to know the concentration of nutrients [9]. The results of these laboratory analyses are compared with the published reference levels at different phenological stages to make sustainable and efficient recommendations on fertilisation. However, leaf ionomic analyses are complex, expensive, and time-consuming and involve high reagent costs and negative environmental impact.

Optical sensors are emerging as a faster and more economical alternative to nutritional diagnosis. These sensors can measure the electromagnetic energy reflected, absorbed or transmitted by vegetation (spectral signature) at different wavelengths. Biotic or abiotic stresses, diseases or nutritional deficiencies affect this energy and the spectral signature. It can therefore be related to crop nutritional status [10,11]. Visible and near-infrared (Vis-NIR) spectroscopy is the most commonly used technology to obtain these measurements [12]. In this context, Vis-NIR spectroscopy has shown potential as a fast, non-destructive method for analysing several plant features [13,14,15,16]. The principle is based on biochemical changes that result in photosynthetic activity, cell structure, and stability of chemical bond variations, promoting changes in reflectance [14].

Several studies have confirmed the potential of spectroscopy to obtain the concentration of nutrients in different crops. Menessatti et al. [17] predicted K and N in citrus leaves with high accuracy using Vis-NIR spectroscopy and leaves from trees with different N treatments. Galvez-Sola et al. [18] determined N, K, Ca, Mg, B, Fe, Cu, Mn, and Zn concentrations in different species of citrus leaves using Fourier Transform NIR (FT-NIR) spectroscopy. In other fruit trees, Phanomsophon et al. [19] calibrated models based on partial least squares (PLS) to predict N and K concentration levels in durian leaves with higher accuracy. In vine leaves, Cuq et al. [20] studied the nutritional status of P, K, Ca, Mg, Mn, Fe, Cu, Zn, and B contents in different vine organs (leaf blades, petioles and berries) using PLS models. The best prediction model was shown for Ca and Mg with R^2^ = 0.88, 0.70, 0.72 and 0.60, 0.72, and 0.80 for limbs, petioles, and berries, respectively. In wheat and oats, N and Mg deficiencies were found to cause a great increase in reflectance in both Vis and NIR spectral ranges, and deficiencies of P and K resulted in a decrease in the 412 to 770 nm range [21]. Additionally, Johnson et al. [22] found that the combination of NIR and mid-infrared (MIR) ranges showed good potential for the determination of both macronutrient (N, P, K, Ca, Mg, and S) and micronutrient (Na, Fe, Mn, B, Cu, Mo, and Zn) concentrations in rice plants (straw and paddy) using PLS. Yarce et al. [23] found high correlations using the NIR region to predict macro and micronutrients (Ca, Mg, N, P, K, Cu, Zn, Mn, and Fe) in sugarcane, and Chen et al. [24] used the same spectral region to predict P. More recent work has been carried out to study the nutrient concentration in persimmon leaves, achieving better results using spectroscopy [25] than hyperspectral imaging [26].

The European Union (EU) is committed to promoting sustainable agriculture and reducing the use of fertilisers by 50% by 2030 as part of its European Green Deal and Farm to Fork strategy. To achieve this goal, developing tools and robust predictive models for nutrient assessment is essential to develop rational fertilisation plans further. Each plant species and variety have its own nutritional needs. Therefore, it is essential to understand the specific nutritional requirements of the variety to ensure optimal plant health and productivity. Few studies conducted to determine nutrients in citrus leaves using Vis-NIR spectroscopy have been found. This work advances the development of a non-destructive tool for the prediction of the foliar concentrations of macro (N, P, K, Ca, Mg) and micronutrients (Na, S, Fe, Cu, Mn, Zn, B) in citrus leaves based on Vis-NIR spectroscopy, using a portable field spectrometer. This tool would boost the establishment of rational fertilisation programmes.

## 2. Materials and Methods

### 2.1. Samples

The study was carried out on eight-year-old trees commercial plot of clementine mandarins (*Citrus clementina* Hort. Ex Tan.) grafted on two rootstocks, Citrus *macrophylla* and Citrange *carrizo*. The plot was located in Almenara (Castellón), Spain (39°44′59.75″ N and 0°13′39.76″ W), on a loam-clay soil with good drainage and a depth greater than 1.5 m with a total area of 72.2 ha. Table 1 shows the amount of nutrients applied in the experimental plot. These doses are recommended for citrus cultivation in Mediterranean growing conditions [8].

Leaf samplings were carried out in June, July, September, October, November 2020 and January 2021 to enhance the variability of foliar nutrient concentrations. Each month, 12 samples of eight spring flush leaves from the non-fruiting shoots were randomly collected and separately bagged. The eight leaves in the same bag were later used for every single ionomic analysis. A total of 592 leaves were collected because the last sampling included 16 more leaves. The samples were transported to the laboratory, washed with deionised water and placed on a paper towel to dry the moisture.

### 2.2. Spectral Acquisition

**Figure 1:**
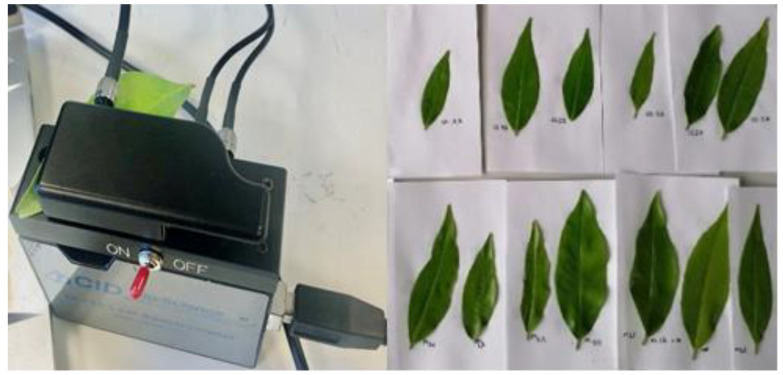
Spectral acquisition of citrus leaves in absorbance mode.

The CI-710 Miniature Leaf Spectrometer (CID Bio-Science, Inc., Camas, WA, USA) was used to record the spectra of the leaves. This fully portable spectrometer can measure Vis-NIR transmittance, absorbance or reflectance spectrum between 345 and 1050 nm. The radiation source emitted consists of a combination of a blue LED and an incandescent lamp, and a clip system protects the measurement area from interference from the environmental light. Measurements were taken in 232 bands in the spectral range 430–1040 nm at intervals of 2.6 nm. The spectral measurements were performed in absorbance mode at two points of each leaf, one near the apex and the other near the petiole. The average spectrum of these two points was obtained. Figure 1 shows the acquisition of the spectrum of the citrus leaf at a point on the leaf, specifically at the apex, in absorbance mode, with the CI-710 spectrometer.

### 2.3. Foliar Ionomic Analysis

After the spectral measurements, the leaves were dried in a forced air oven at 65 °C for a minimum of 72 h and ground to 1 mm with a water-cooled mill (IKA M 20, IKA Labortechnik, Staufen, Germany).

Ionomic analyses were then performed using eight leaves for every single analysis. The Kjeldahl method [27] was used for organic N analysis using a Tecator Kjeltec 8200 TM Digestor (FOSS, Hillerød, Denmark). The other macro and microelements were determined by an inductively coupled plasma optical emission spectrometry iCAP 7000 Plus Series ICP-OES (Thermo Scientific, Waltham, MA USA). Nutrient extraction was performed by wet digestion using a microwave (Milestone ETHOS UP, Sorisole, BG. Italy). For this, 0.200 g of the crushed dry samples were weighed, and 4 mL of Milli-Q, 4 mL of nitric acid (HNO₃) and 2 mL of hydrogen peroxide (H_2_O_2_) were added to each sample. The tubes were kept at 200 °C for 15 to 20 min. Once digestion had finished, the extracts were diluted in 25 mL tubes and micronutrient concentrations were analysed in the ICP. The concentration was calculated through Equation (1).

Micronutrient concentration = ((*a* − *b*) × *V*)/*P* (1)

where *a* was the concentration of Fe, Zn, Mn, Cu, and B in the solution of the digestion of the foliar sample (mg·L^−1^); *b* was the concentration of these nutrients in the blank (mg·L^−1^); *V* was the final volume of digestion (25 mL); and *P* was the dry weight of the sample digested.

An aliquot of 0.5 mL was taken from the extraction solution to determine the macronutrients and made up to 10 mL with Milli-Q water. The concentration was calculated using Equation (2).

Macronutrient concentration = ((*a* − *b*) × *V* × *d*))/(*P* × 1000) (2)

where *a* was the concentration of P, K, Mg, Ca, and S in the aliquot from the digestion of the sample (mg L^−1^); *b* was the concentration of these nutrients in the blank (mg L^−1^); *V* was the final volume of digestion (25 mL); *P* was the dry weight of the sample digested; and *d* was the dilution factor.

Mo, Cl, and Ni were not included in the analysis since these nutrients were recently considered essential elements in plants [28], and, hence, the focus was placed on the nutrients with reference values for nutritional diagnosis that had already been established.

### 2.4. Chemometric Analysis

The two spectra obtained from each sample were averaged to obtain a single value per leaf and correlated with the nutrient concentrations determined by ionomic analysis.

Five pre-treatments were applied to the spectra. Mean centre (MC) was used to centre each variable by subtracting the mean of all the elements of that variable [29]. Savitzky-Golay (SG) smoothing [30] was applied to reduce random noise and increase the signal-to-noise ratio. A reduction in dispersion was performed through Standard Normal Variate (SNV) [31], while the first (1D) and the second derivatives (2D) were used to eliminate constant baseline offsets and offsets that vary linearly with wavelength [32]. Models combining MC with the other techniques were trained and tested.

PLS-regression (PLS-R) is a common technique to establish a correlation between sample spectra and the properties of interest, such as nutrient concentration [33]. In this work, PLS-R models were trained to predict the nutrient content in citrus leaves [34]. A separate model was developed for each nutrient through the creation of a table with the leaf samples as rows and pre-treated spectra (X-variables) as columns. An additional column was added to each table to include the actual nutrient concentration values obtained from ionomic analyses, which served as the variable to be predicted (Y-variable).

Samples were randomly divided into a training set (75%) for calibration and an independent test set (25%) for external validation. We checked that there were no statistical differences in the nutrient concentrations of both tests. The training set was used for calibration and cross-validation (CV), while the test set was for external validation. Parameter optimisation is essential to improve the efficiency and accuracy in the development of models [35,36]. CV was employed to determine the optimal number of PLS-R latent variables (LV) and estimate the model uncertainty in the training set [37]. The model with the lowest root-mean-square error (RMSE) and the highest coefficient of determination (R^2^) in the test of the model was selected, along with the LV used to calibrate a robust model. LV refers to a series of factors used to build a reliable model, and a smaller number of components with a lower error indicate a higher level of prediction accuracy [38].

In addition, a study was also conducted to determine whether a subset of wavelengths is important for predicting nutrients. In this work, the weighted regression coefficients (BW) of the PLS-R model were used to know the wavelengths of interest for the prediction of the micro and macro elements. This method measures the association between each wavelength and the content of the element under study, where wavelengths with large absolute BW coefficient values are the most important in the model [39]. The BW coefficients were calculated directly from the PLS loadings corresponding to the model with the optimum number of LV [40]. This selection was only applied to the PLS-R models with an R^2^ higher than 0.60.

## 3. Results and Discussion

### 3.1. Descriptive Statistics of the Foliar Macro and Micronutrient Concentrations

Table 2 shows the descriptive statistical values of foliar macro and micronutrient concentrations of the leaves taken throughout the crop cycle, as determined by ionomic procedures. This table summarises the values found for all samples, but both the calibration and validation sets had very similar values and distribution for the concentrations. The values obtained for the sampling carried out in November are shown in brackets since it is considered the optimal date for the nutritional diagnosis of citrus. N and Cu nutrients were found to be deficient. The value obtained for P was low. Nutrients such as K, Na, S, Fe, Mn, Zn, and B yielded an optimum value. The results obtained for Ca and Mg were high. The classification was developed according to the reference parameters described in Quiñones et al. [8].

### 3.2. PLS-R Models for Macro and Micronutrients Estimation

Table 3 presents the predictive results for each element using PLS-R with the optimal spectra pre-treatment. The macronutrients P, K, and Ca showed the highest accuracy for the calibration of the models using CV. The P model was calibrated using 10 LVs and MC, with an R^2^ of 0.66 being obtained. The K model was calibrated using 12 LVs and MC + SNV, with an R^2^ of 0.58, while Ca was calibrated using 7 LVs and MC + 1D, an R^2^ of 0.63 being obtained. Using the test set, an R^2^ of 0.60 was achieved for P, and the K model obtained an R^2^ of 0.63. However, the R^2^ for Ca was lower (0.53). In the case of N, the performance of the model was lower, with an R^2^ of 0.57 being obtained.

Regarding the micronutrients, the model for B was the most accurate in calibration and testing. This model was calibrated using 7 LV and MC + 1D, an R^2^ of 0.64 being obtained in the CV and an R^2^ of 0.69 in the test. In all cases, a relatively low RMSE was achieved.

The results for macronutrient prediction were particularly noteworthy for P and K: R^2^ = 0.60 using MC and R^2^ = 0.63 using MC + SNV, respectively. B had the highest prediction accuracy for micronutrients, with an R^2^ = 0.69 obtained using an MC + 1D pre-treatment. The models were less accurate for the rest of the micro and macronutrients (R^2^ < 0.60).

Comparable results were achieved in previous scientific literature, but this study has unique features that make the outcomes closer to natural crop stages. Osco et al. [41] predicted the concentration of macro and micronutrients in ‘Valencia’ orange leaves using a handheld spectroradiometer in the spectral range of 380 to 1020 nm. For the macronutrients, the R^2^ range was between 0.62 and 0.90. However, the study was based on a limited test set of 32 leaves and had several misconceptions. The methods used were primarily for classification rather than regression, making it unclear how the coefficients of determination were obtained.

Additionally, the number of leaves required for chemical analysis was not stated, and only one leaf might not suffice to obtain the minimum amount required as with traditional methods. Galvez-Sola et al. [18] used FT-NIR (830–2600 nm) to predict macronutrients for six different citrus tree species, achieving R^2^ values ranging from 0.88 to 0.99. However, the spectral measurements were conducted on powdered leaf samples, thus forfeiting some of the primary advantages of these methods, such as simplicity and non-destructiveness, and avoiding the need to process the samples. Since the different citrus species exhibit varying nutrient concentrations, the results could be influenced by intrinsic species-specific factors. Obtaining practical results would therefore require knowledge of the predictions for each species independently. Menessatti et al. [17] employed Vis-NIR spectroscopy to determine macronutrient levels in 20 Tarocco leaves. They observed high R^2^ for all nutrients except for P. However, the experimental setup involved using a randomised block design with five rates of N input at varying levels, ranging from 0 to 800 g N per tree per year, from no N to twice the typical recommendations. As a result, the models could predict extreme cases of nutrient deficiency or excess, but their ability to accurately predict or differentiate nutrient concentrations under more typical fertilisation rates was not demonstrated. On the contrary, the present study successfully captured the natural variability of nutrient values observed in the field throughout the season under commercial recommendations.

### 3.3. Evaluation of Relevant Wavelengths for Prediction

**Figure 2:**
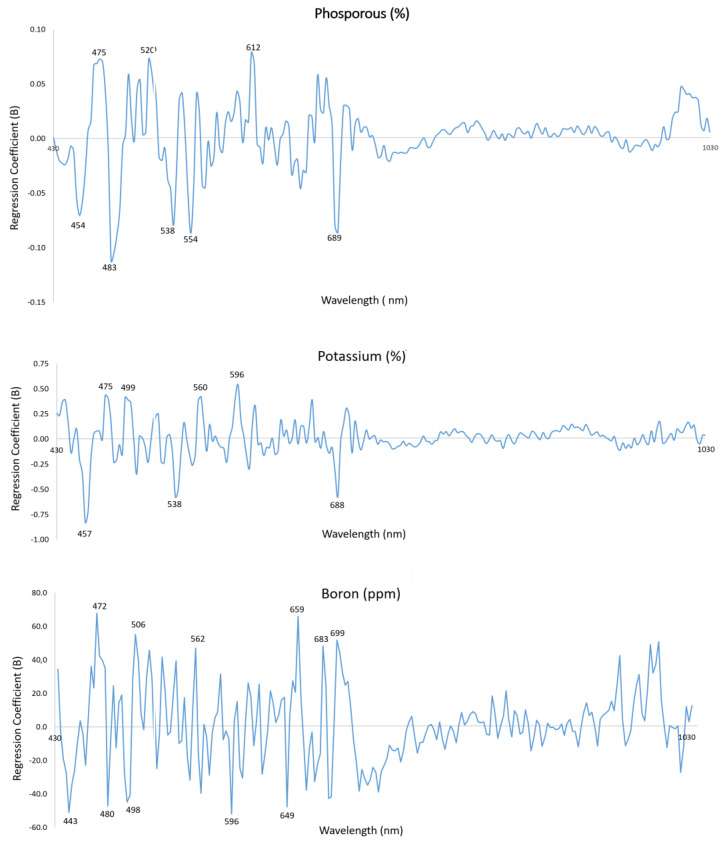
BW coefficients from PLS-R models of P, K, and B.

Figure 2 shows the BW coefficients with the associated wavelengths for the most accurate models (P, K and B). The important wavelengths selected for P, K, and B were close to 440–530 nm and 560–690 nm. For P, the selected wavelengths, in order of importance, were 483, 554, 689, 538, 454, 475, 520, and 612 nm. For K, they were 457, 538, 596, 688, 475, 560, and 499 nm, and for B, they were 472, 659, 506, 596, 443, 649, 683, 480, 562, 498, and 699 nm.

The wavelengths selected were all situated in the visible region (430–750 nm), which is related to photosynthetic pigments that absorb about 90% or more of the incoming light [42]. In contrast, there are no strongly absorbing molecules in the NIR, so plants refract or transmit all but about 10% of the incoming radiation in this region [43]. This is also compatible with the fact that deficiency or excess of some nutrients can affect pigment accumulation in leaves, which has an influence on the absorption or refraction of specific wavelengths of visible light. These photosynthetic pigments in the leaves are mainly chlorophylls [44] and carotenoids [45], which play a crucial role in plant photosynthesis. In this process, the plant forms sugars from the energy received from sunlight and CO_2_ that the plant absorbs. Chlorophylls are divided into chlorophyll a (Chl-a) and chlorophyll b (Chl-b), which are responsible for the characteristic green colour of leaves with an absorption peak around 450 and 680 nm. On the other hand, carotenoids are divided into carotene a, carotene b and xanthophylls, and exhibit strong light absorption in the blue region of the spectrum (450–500 nm) [46].

Few studies have identified which individual wavelengths are the most important in predicting macro and micronutrients in citrus leaves. Osco et al. [41] used a Vis-NIR spectroradiometer to identify the optimal wavelengths and observed that all bands selected to predict P were found in the visible region, while for K, they were located only in the NIR region. In apple leaves, Azadnia et al. [47] used Vis-NIR spectroscopy and variable importance in projection (VIP) scores to select the most important wavelengths for P and K. For P, all the selected bands were also located mainly in the visible region (between 575 and 700 nm), except for one that was selected around 970 nm. In the case of K, they found six spectral regions in the visible (between 505 and 700 nm) and NIR (between 920 and 965 nm).

This technology provides a detailed analytical view of nutrient content in plants for rapid non-destructive estimates of macro and micronutrients in leaves, which will pave the way to the planning of better and more efficient fertilisation systems under a precision agriculture strategy. However, little research has been performed on using alternative techniques, such as Vis-NIR spectroscopy, for macro and micronutrient prediction in orchard crops. The results achieved indicated that it is possible to perform relatively accurate prediction for some nutrients such as P, K, and B, while it is necessary to continue working to achieve better models for the others. Important wavelengths have been found only in the visible part of the spectrum, which can guide future work to advance in this direction. Chemometric methods are possibly the primary approach to analyze spectral data. However, algorithms based on deep learning approaches could be further trained and optimized to achieve robust results capturing the variability found in the samples throughout the entire season. Moreover, this work also advances to achieve future field measurements for macro and micronutrient prediction. Currently, there are no portable spectral devices aimed at estimating nutrient concentration, and the closest solutions come from chlorophyll meters used to estimate N, with limited success.

## 4. Conclusions

This work has studied the potential of Vis-NIR spectroscopy to predict the concentration of nutrients in Clementina de Nules citrus leaves through a vegetative cycle as a faster and non-destructive alternative to foliar ionomic analyses. The results showed a good ability (R^2^ > 0.60) to estimate the concentrations of P, K, and B with relatively low RMSE for the independent prediction set. The other nutrients studied were estimated with relatively lower performance. Effective wavelengths were found in the visible region for P, K, and B using the BW coefficients, which suggest that this region contains the most relevant information for nutrient prediction. Hence, future works should focus on it.

## Figures and Tables

**Table 1:** Fertiliser units (kg/ha) applied to the experimental plot.

Annual Dose Chemical Compound (kg/ha)	
N	240
P_2_O_5_	80
K_2_O	140
MgO	180
Fe	1

**Table 2:** Ionomic analysis of the citrus leaves. Concentrations are expressed in % for macronutrients (N, P, K, Ca, Mg, Na, S) and mg/kg for micronutrients (Fe, Cu, Mn, Zn, B) based on the dry matter weight.

	N	P	K	Ca	Mg	Na	S	Fe	Cu	Mn	Zn	B
November	1.94	0.11	0.83	5.65	0.52	0.03	0.33	83.13	3.03	25.1	26.7	46.61
Mean	2.10 (D)	0.13 (L)	1.00 (O)	4.01 (H)	0.41 (H)	0.03 (O)	0.28 (O)	72.26 (O)	3.95 (D)	22.32 (O)	28.51 (O)	41.88 (O)
Max	2.78	0.22	1.71	6.57	0.68	0.06	0.37	149.9	14.8	54.93	57.64	94.9
Min	1.48	0.05	0.36	1.05	0.12	0.01	0.16	28.9	0.75	6.34	3.48	21.57
SD	0.29	0.04	0.35	1.36	0.13	0.01	0.05	27.65	2.77	11.44	14.25	12.12
Median	2.06	0.14	0.96	4.18	0.41	0.03	0.29	65.75	3.19	20.98	27.48	41.13

SD: standard deviation; D = deficient; L = low; O = optimum; H = high

**Table 3:** Results for calibration, cross-validation, and test sets using PLS-R.

Nutrient	Pre-Treatment	LVs	Calibration		Cross-Validation		Test Set	
			RMSE	R^2^	RMSE	R^2^	RMSE	R^2^
N	MC	10	0.18	0.58	0.18	0.55	0.19	0.57
P	MC	10	0.02	0.69	0.02	0.66	0.02	0.60
K	MC + SNV	12	0.21	0.65	0.23	0.58	0.22	0.63
Ca	MC + 1D	7	0.65	0.67	0.69	0.63	0.73	0.53
Mg	Raw	9	0.08	0.52	0.08	0.47	0.08	0.47
S	MC	11	0.02	0.52	0.03	0.48	0.03	0.44
Fe	MC	7	24.93	0.48	24.93	0.46	24.39	0.48
Cu	Raw	9	0.93	0.33	0.95	0.29	0.93	0.31
Mn	MC + SNV	12	7.73	0.53	8.42	0.44	8.07	0.49
Zn	MC + 1D	7	9.94	0.50	10.52	0.44	10.25	0.46
B	MC + 1D	7	5.26	0.70	5.75	0.64	5.83	0.69

LV: Latent variables; RMSE: Root mean square error; MC: mean centre; SNV: standard normal variate; 1D: first derivative.

## Data Availability

Not applicable.

## Abbreviations

Artificial neural networks (ANN)	Molybdenum (Mo)
Boron (B)	Multiple linear regression (MLR)
Calcium (Ca)	Near infrared spectroscopy (NIRS)
Carbon (C)	Nitrogen (N)
Carbon dioxide (CO_2_)	Nickel (Ni)
Chlorine (Cl)	Oxygen (O)
Chlorophyll a (Chl-a)	Partial least squares (PLS)
Chlorophyll b (Chl-b)	PLS-regression (PLS-R)
Copper (Cu)	Phosphorus (P)
Cross-validation (CV)	Principal component regression (PCR)
Emission spectrometry (ICP-OES)	Random forest (RF)
First derivative (1D)	Root mean square error (RMSE)
Fourier Transform NIR (FT-NIR)	Standard normal variate (SNV)
Hydrogen (H)	Sulphur (S)
Iron (Fe)	Support vector machine (SVM)
Latent variables (LV)	Variable importance in projection (VIP)
Magnesium (Mg)	Visible (Vis)
Manganese (Mn)	Weight regression coefficients (BW)
Mean centre (MC)	Zinc (Zn)

## References

[1] Food and Agriculture Organization (FAO) Citrus Fruit. Fresh and Processed Statistical Bulletin 2020. Market and Trade Commodities. https://www.fao.org/markets-and-trade/commodities/citrus/en.

[2] United States Department of Agriculture (USDA) Citrus: World Market and Trade. Office of Global Analysis. Foreign Agriculture Service. https://www.fas.usda.gov/data/citrus-world-markets-and-trade.

[3] United States Department of Agriculture (USDA) Citrus Annual. Foreign Agricultural Service. https://apps.fas.usda.gov/newgainapi/api/Report/DownloadReportByFileName?fileName=Citrus%20Annual_Madrid_European%20Union_E42023-0001.pdf.

[4] Marschner P. (2012). Rhizosphere Biology. Marschner’s Mineral Nutrition of Higher Plants.

[5] Millard P. (1996). Ecophysiology of the Internal Cycling of Nitrogen for Tree Growth. Z. Pflanzenernahr. Bodenkd..

[6] Bassi D., Menossi M., Mattiello L. (2018). Nitrogen Supply Influences Photosynthesis Establishment along the Sugarcane Leaf. Sci. Rep..

[7] Payne R.J., Dise N.B., Field C.D., Dore A.J., Caporn S.J.M., Stevens C.J. (2017). Nitrogen Deposition and Plant Biodiversity: Past, Present, and Future. Front. Ecol. Environ..

[8] Quinones A., Martínez-Alcántara B., Primo-Millo E., Legaz F. (2010). Abonado de Los Cítricos. Guía Práctica de la Fertilización Racional de los Cultivos en España.

[9] Shenk J.S., Westerhaus M.O., Hoover M.R. (1979). Analysis of Forages by Infrared Reflectance. J. Dairy Sci..

[10] Mcvicar T.R., Briggs P.R., King E.A., Raupach M.R. (2003). A Review of Predictive Modelling from a Natural Resource Management Perspective: The Role of Remote Sensing of the Terrestrial Environment By CSIRO.

[11] Carter G.A. (1998). Reflectance Wavebands and Indices for Remote Estimation of Photosynthesis and Stomatal Conductance in Pine Canopies. Remote Sens. Environ..

[12] Walsh K.B., Blasco J., Zude-Sasse M., Sun X. (2020). Visible-NIR ‘Point’ Spectroscopy in Postharvest Fruit and Vegetable Assessment: The Science behind Three Decades of Commercial Use. Postharvest Biol. Technol..

[13] Guo T., Tan C., Li Q., Cui G., Li H. (2019). Estimating Leaf Chlorophyll Content in Tobacco Based on Various Canopy Hyperspectral Parameters. J. Ambient. Intell. Humaniz. Comput..

[14] Ling B., Goodin D.G., Raynor E.J., Joern A. (2019). Hyperspectral Analysis of Leaf Pigments and Nutritional Elements in Tallgrass Prairie Vegetation. Front. Plant Sci..

[15] Rodrigues M., Nanni M.R., Cezar E., dos Santos G.L.A.A., Reis A.S., de Oliveira K.M., de Oliveira R.B. (2020). Vis–NIR Spectroscopy: From Leaf Dry Mass Production Estimate to the Prediction of Macro- and Micronutrients in Soybean Crops. J. Appl. Remote Sens..

[16] Dos Santos G.L.A.A., Reis A.S., Besen M.R., Furlanetto R.H., Rodrigues M., Crusiol L.G.T., de Oliveira K.M., Falcioni R., de Oliveira R.B., Batista M.A. (2023). Spectral Method for Macro and Micronutrient Prediction in Soybean Leaves Using Interval Partial Least Squares Regression. Eur. J. Agron..

[17] Menesatti P., Pallottino F., Antonucci F., Roccuzzo G., Intrigliolo F., Costa C. (2012). Non-Destructive Proximal Sensing for Early Detection of Citrus Nutrient and Water Stress. Advances in Citrus Nutrition.

[18] Galvez-Sola L., García-Sánchez F., Pérez-Pérez J.G., Gimeno V., Navarro J.M., Moral R., Martínez-Nicolás J.J., Nieves M. (2015). Rapid Estimation of Nutritional Elements on Citrus Leaves by near Infrared Reflectance Spectroscopy. Front. Plant Sci..

[19] Phanomsophon T., Jaisue N., Tawinteung N., Khurnpoon L., Sirisomboon P. (2022). Classification of N, P, and K Concentrations in Durian (Durio Zibethinus Murray CV. Mon Thong) Leaves Using near-Infrared Spectroscopy. Eng. Appl. Sci. Res..

[20] Cuq S., Lemetter V., Kleiber D., Levasseur-Garcia C. (2020). Assessing Macro- (P, K, Ca, Mg) and Micronutrient (Mn, Fe, Cu, Zn, B) Concentration in Vine Leaves and Grape Berries of Vitis Vinifera by Using near-Infrared Spectroscopy and Chemometrics. Comput. Electron. Agric..

[21] Ayala-Silva T., Beyl C.A. (2005). Changes in Spectral Reflectance of Wheat Leaves in Response to Specific Macronutrient Deficiency. Adv. Space Res..

[22] Johnson J.M., Sila A., Senthilkumar K., Shepherd K.D., Saito K. (2021). Application of Infrared Spectroscopy for Estimation of Concentrations of Macro- and Micronutrients in Rice in Sub-Saharan Africa. Field Crops Res..

[23] Yarce C.J., Rojas G. (2012). Near Infrared Spectroscopy for the Analysis of Macro and Micro Nutrients in Sugarcane Leaves. Zuckerindustrie.

[24] Chen M., Glaz B., Gilbert R.A., Daroub S.H., Barton F.E., Wan Y. (2002). Near-Infrared Reflectance Spectroscopy Analysis of Phosphorus in Sugarcane Leaves. Agron. J..

[25] Acosta M., Visconti F., Quiñones A., Blasco J., de Paz J.M. (2023). Estimation of Macro and Micronutrients in Persimmon (*Diospyros Kaki* L.) cv. ‘Rojo Brillante’ Leaves through Vis-NIR Reflectance Spectroscopy. Agronomy.

[26] Acosta M., Rodríguez-Carretero I., Blasco J., de Paz J.M., Quiñones A. (2023). Non-Destructive Appraisal of Macro- and Micronutrients in Persimmon Leaves Using Vis/NIR Hyperspectral Imaging. Agriculture.

[27] Bodenkunde I. (1982). Inorganic Forms of Nitrogen in Soil. Nitrogen Agric. Soils.

[28] Marschner H. (1985). Mineral Nutrition of Higher Plants.

[29] Ulissi V., Antonucci F., Benincasa P., Farneselli M., Tosti G., Guiducci M., Tei F., Costa C., Pallottino F., Pari L. (2011). Nitrogen Concentration Estimation in Tomato Leaves by VIS-NIR Non-Destructive Spectroscopy. Sensors.

[30] Savitzky A., Golay M.J.E. (1964). Smoothing and Differentiation of Data by Simplified Least Squares Procedures. Anal. Chem..

[31] Barnes R.J., Dhanoa M.S., Lister S.J. (1989). Standard Normal Variate Transformation and De-Trending of Near-Infrared Diffuse Reflectance Spectra. Appl. Spectrosc..

[32] Alchanatis V., Schmilovitch Z., Meron M. (2005). In-Field Assessment of Single Leaf Nitrogen Status by Spectral Reflectance Measurements. Precis. Agric..

[33] Furlanetto R.H., Moriwaki T., Falcioni R., Pattaro M., Vollmann A., Sturion Junior A.C., Antunes W.C., Nanni M.R. (2020). Hyperspectral Reflectance Imaging to Classify Lettuce Varieties by Optimum Selected Wavelengths and Linear Discriminant Analysis. Remote Sens. Appl. Soc. Environ..

[34] Lindgren F., Geladi P., Wold S. (1993). The Kernel Algorithm for PLS. J. Chemom..

[35] Dimov I., Georgieva R., Todorov V. (2015). Balancing of Systematic and Stochastic Errors in Monte Carlo. Algorithms for Integral Equations.

[36] Todorov V., Dimov I. (2020). Efficient Stochastic Approaches for Multidimensional Integrals in Bayesian Statistics.

[37] Cawley G.C., Talbot N.L.C. (2003). Efficient Leave-One-out Cross-Validation of Kernel Fisher Discriminant Classifiers. Pattern Recognit..

[38] Wold S., Sjöström M., Eriksson L. (2001). PLS-Regression: A Basic Tool of Chemometrics. Chemom. Intell. Lab. Syst..

[39] Mehmood T., Liland K.H., Snipen L., Sæbø S. (2012). A Review of Variable Selection Methods in Partial Least Squares Regression. Chemom. Intell. Lab. Syst..

[40] Frenich A.G., Jouan-Rimbaud D., Massart D.L., Kuttatharmmakul S., Galera M.M., Vidal J.L.M. (1995). Wavelength Selection Method for Multicomponent Spectrophotometric Determinations Using Partial Least Squares. Analyst.

[41] Osco L.P., Ramos A.P.M., Pinheiro M.M.F., Moriya É.A.S., Imai N.N., Estrabis N., Ianczyk F., de Araújo F.F., Liesenberg V., de Castro Jorge L.A. (2020). A Machine Learning Framework to Predict Nutrient Content in Valencia-Orange Leaf Hyperspectral Measurements. Remote Sens..

[42] Gates D. (1965). Energy, Plants and Ecology. Ecology.

[43] Jacquemoud S.U.S. Modeling Leaf Optical Properties. Photobiological. Photobiological Sciences Online. Environmental Photobiology. http://www.photobiology.info/#environ.

[44] Sonobe R., Sano T., Horie H. (2018). Using Spectral Reflectance to Estimate Leaf Chlorophyll Content of Tea with Shading Treatments. Biosyst. Eng..

[45] Gitelson A.A., Zur Y., Chivkunova O.B., Merzlyak M.N. (2002). Assessing Carotenoid Content in Plant Leaves with Reflectance Spectroscopy. Photochem. Photobiol..

[46] Demmig-Adams B., Gilmore A.M., Iii W.W.A. (1996). In Vivo Functions of Carotenoids in Higher Plants. FASEB J..

[47] Azadnia R., Rajabipour A., Jamshidi B., Omid M. (2023). New Approach for Rapid Estimation of Leaf Nitrogen, Phosphorus, and Potassium Contents in Apple-Trees Using Vis/NIR Spectroscopy Based on Wavelength Selection Coupled with Machine Learning. Comput. Electron. Agric..

